# Supplementary material for: Strip cropping shows promising increases in ground beetle community diversity compared to monocultures
Source: eLife. 2025 Sep 23;14:RP104762. doi: 10.7554/eLife.104762 (PMC12456951; doi:10.7554/eLife.104762)
Supplement: Figure 2—source data 1. — The first value indicates the estimated mean, in brackets the confidence interval and letters indicate significant difference between monocultures and strips per location. A stripe indicates that there was no significant difference for the genus for that location. Dark cells indicate that two or fewer individuals were found at this location, and the location was excluded from the model. At Wageningen, we only found a few Anchomenus in the monoculture, whereas we found none in the strip-cropped field. As there was no variation in the strip-cropped field, this location could not be included in the model for this genus, and the mean here indicates the actual mean. The model for Pterostichus did not fit well when the data from 2020 Almere were included, as catches were much higher in this year than in the other years. Therefore, we conducted separate analyses for 2020 and 2021–2022. [file elife-104762-fig2-data1.docx]

**Figure 2-source data 1.** Abundances of the twelve most abundant ground beetle genera in monoculture and strip cropping fields in four locations. The first value indicates the estimated mean, in brackets the confidence interval and letters indicate significant difference between monocultures and strips per location. A strip indicates that there was no significant difference for the genus for that location. Dark cells indicate that two or less individuals were found at this location, and the location was excluded from the model. At Wageningen, we only found few *Anchomenus* in the monoculture, whereas we found none in the strip cropped field. As there was no variation in the strip cropped field, this location could not be included in the model for this genus and the mean here indicates the actual mean. The model for Pterostichus did not fit well when the data from 2020 Almere were included, as catches were much higher in this year than in the other years. Therefore, we conducted separate analyses for 2020 and 2021-2022.

|  | **Almere** | | **Lelystad** | | **Valthermond** | | **Wageningen** | |
| --- | --- | --- | --- | --- | --- | --- | --- | --- |
| **Genus** | **Mono** | **Strip** | **Mono** | **Strip** | **Mono** | **Strip** | **Mono** | **Strip** |
| *Amara* | 0.44  (0.17-1.15)  B | 0.25  (0.09-0.67)  A |  |  | 0.11  (0.02-0.57)  - | 0.13  (0.03-0.55)  - | 0.51  (0.20-1.33)  - | 0.76  (0.29-2.00)  - |
| *Anchomenus* | 0.06  (0.02-0.20)  A | 0.20  (0.06-0.64)  B |  |  | 0.91  (0.18-4.70)  - | 1.89  (0.41-8.78)  - | 0.04  N/A | 0.00  N/A |
| *Bembidion* | 2.91  (2.07-4.10)  A | 4.80  (3.44-6.70)  B | 6.32  (3.30-12.1)  - | 4.35  (2.12-8.93)  - | 0.46  (0.20-1.06)  - | 0.52  (0.28-0.98)  - | 0.53  (0.35-0.81)  - | 0.56  (0.34-0.91)  - |
| *Blemus* | 0.15  (0.04-0.65)  - | 0.12  (0.03-0.51)  - | 0.73  (0.16-3.36)  - | 0.41  (0.08-2.03)  - |  |  |  |  |
| *Calathus* |  |  |  |  | 2.24  (1.03-4.89)  B | 1.22  (0.57-2.60)  A | 0.50  (0.27-0.93)  - | 0.44  (0.22-0.85)  - |
| *Clivina* | 0.18  (0.08-0.40)  - | 0.15  (0.06-0.34)  - | 0.18  (0.05-0.62)  - | 0.05  (0.01-0.33)  - | 0.10  (0.03-0.42)  - | 0.02  (0.00-0.14)  - | 0.18  (0.08-0.39)  - | 0.11  (0.04-0.28)  - |
| *Harpalus* | 11.2  (6.50-19.3)  A | 15.1  (8.75-26.0)  B | 0.52  (0.21-1.32)  - | 0.62  (0.23-1.68  - | 5.04  (2.51-10.1)  - | 4.40  (2.30-8.41)  - | 4.43  (2.58-7.60)  A | 6.12  (3.50-10.7)  B |
| *Loricera* | 0.05  (0.01-0.32)  - | 0.02  (0.00-0.17)  - | 0.27  (0.03-2.89)  - | 0.14  (0.01-1.92)  - | 0.24  (0.03-1.79)  - | 0.08  (0.01-0.60)  - | 0.03  (0.00-0.19)  - | 0.06  (0.01-0.41)  - |
| *Nebria* | 0.70  (0.23-2.17)  A | 1.30  (0.42-4.00)  B |  |  |  |  | 0.10  (0.03-0.31)  - | 0.06  (0.02-0.24)  - |
| *Poecilus* | 19.1  (13.8-26.5)  - | 21.8  (15.7-30.3)  - | 1.83  (0.98-3.39)  - | 1.54  (0.76-3.11)  - | 1.03  (0.52-2.04)  - | 0.87  (0.47-1.61)  - | 0.17  (0.10-0.29)  - | 0.18  (0.10-0.34)  - |
| *2020*  *Pterostichus*  *2021-22* | 434  (325-581)  B  47.2  (31.2-71.5)  - | 307  (230-411)  A  64.1  (41.6-98.7)  - | 35.1  (16.9-72.6)  - | 30.0  (13.3-68.0)  - | 3.93  (2.09-7.41)  B | 1.70  (1.00-2.88)  A | `3.02  (2.01-4.55)  - | 2.49  (1.58-3.91)  - |
| *Trechus* | 1.25  (0.69-2.26)  B | 0.73  (0.40-1.33)  A | 3.54  (1.37-9.12)  B | 0.91  (0.30-2.79)  A |  |  | 0.55  (0.31-0.98)  - | 0.32  (0.16-0.64)  - |
