# Supplementary material for: Strip cropping shows promising increases in ground beetle community diversity compared to monocultures
Source: eLife. 2025 Sep 23;14:RP104762. doi: 10.7554/eLife.104762 (PMC12456951; doi:10.7554/eLife.104762)
Supplement: Supplementary file 1. [file elife-104762-supp1.docx]

**Supplementary file 1.** All ground beetle species found among the four locations, and their species codes as used in several supplementary figures.

| **Subfamily** | **Genus** | **Species** | **Species code** |
| --- | --- | --- | --- |
| Carabinae | Carabus | *Carabus granulatus* | CaraGran |
|  | Nebria | *Nebria brevicollis* | NebrBrev |
|  |  | *Nebria salina* | NebrSali |
|  | Notiophilus | *Notiophilus aquaticus* | NotiAqua |
|  |  | *Notiophilus biguttatus* | NotiBigu |
|  |  | *Notiophilus palustris* | NotiPalu |
| Harpalinae | Acupalpus | *Acupalpus meridianus* | AcupMeri |
|  | Agonum | *Agonum muelleri* | AgonMuel |
|  | Amara | *Amara aenea* | AmarAene |
|  |  | *Amara anthobia* | AmarAnth |
|  |  | *Amara apricaria* | AmarApri |
|  |  | *Amara aulica* | AmarAuli |
|  |  | *Amara bifrons* | AmarBifr |
|  |  | *Amara communis* | AmarComm |
|  |  | *Amara consularis* | AmarCons |
|  |  | *Amara famelica* | AmarFame |
|  |  | *Amara familiaris* | AmarFami |
|  |  | *Amara fulva* | AmarFulv |
|  |  | *Amara ovata* | AmarOvat |
|  |  | *Amara plebeja* | AmarPleb |
|  |  | *Amara similata* | AmarSimi |
|  |  | *Amara spreta* | AmarSpre |
|  |  | *Amara tibialis* | AmarTibi |
|  | Anchomenus | *Anchomenus dorsalis* | AnchDors |
|  | Anisodactylus | *Anisodactylus binotatus* | AnisBino |
|  | Badister | *Badister bullatus* | BadiBull |
|  |  | *Badister sodalist* | BadiSoda |
|  | Bradycellus | *Bradycellus harpalinus* | BradHarp |
|  | Calathus | *Calathus cinctus* | CalaCinc |
|  |  | *Calathus erratus* | CalaErra |
|  |  | *Calathus fuscipes* | CalaFusc |
|  |  | *Calathus melanocephalus* | CalaMela |
|  |  | *Calathus rotundicollis* | CalaRotu |
|  | Harpalus | *Harpalus affinis* | HarpAffi |
|  |  | *Harpalus distinguendus* | HarpDist |
|  |  | *Harpalus griseus* | HarpGris |
|  |  | *Harpalus rubripes* | HarpRubr |
|  |  | *Harpalus rufipes* | HarpRufi |
|  |  | *Harpalus signaticornis* | HarpSign |
|  |  | *Harpalus tardus* | HarpTard |
|  | Microlestes | *Microlestes minutulus* | MicrMinu |
|  | Oxypselaphus | *Oxypselaphus obscurus* | OxypObsc |
|  | Panagaeus | *Panagaeus bipustulatus* | PanaBipu |
|  | Poecilus | *Poecilus cupreus* | PoecCupr |
|  |  | *Poecilus versicolor* | PoecVers |
|  | Pterostichus | *Pterostichus anthracinus* | PterAnth |
|  |  | *Pterostichus melanarius* | PterMela |
|  |  | *Pterostichus niger* | PterNige |
|  |  | *Pterostichus strenuus* | PterStre |
|  |  | *Pterostichus vernalis* | PterVern |
|  | Stenolophus | *Stenolophus teutonus* | StenTeut |
|  | Stomis | *Stomis pumicatus* | StomPumi |
|  | Syntomus | *Syntomus truncatellus* | SyntTrun |
| Loricerinae | Loricera | *Loricera pilicornis* | LoriPili |
| Scaritinae | Broscus | *Broscus cephalotes* | BrosCeph |
|  | Clivina | *Clivina collaris* | ClivColl |
|  |  | *Clivina fossor* | ClivFoss |
|  | Dyschirius | *Dyschirius globosus* | DyscGlob |
| Trechinae | Asaphidion | *Asaphidion flavipes* | AsapFlav |
|  | Bembidion | *Bembidion aenea* | BembAene |
|  |  | *Bembidion biguttatum* | BembBigu |
|  |  | *Bembidion femoratum* | BembFemo |
|  |  | *Bembidion lampros* | BembLamp |
|  |  | *Bembidion lunulatum* | BembLunu |
|  |  | *Bembidion obtusum* | BembObtu |
|  |  | *Bembidion proprans* | BembProp |
|  |  | *Bembidion quadrimaculatum* | BembQuadrim |
|  |  | *Bembidion tetracolum* | BembTetr |
|  | Blemus | *Blemus discus* | BlemDisc |
|  | Trechoblemus | *Trechoblemus micros* | TrecMicr |
|  | Trechus | *Trechus obtusus* | TrecObtu |
|  |  | *Trechus quadristriatus* | TrecQuad |
