# Supplementary material for: Strip cropping shows promising increases in ground beetle community diversity compared to monocultures
Source: eLife. 2025 Sep 23;14:RP104762. doi: 10.7554/eLife.104762 (PMC12456951; doi:10.7554/eLife.104762)
Supplement: Supplementary file 2. — The number of year series is given per location in brackets. For some locations, ground beetles were identified up to genus level, these are underlined. ‘N/A’ indicates that this taxa was identified to a different taxonomic level for the specific location. Locations are indicated with abbreviations (Al=Almere; Le = Lelystad; Va = Valthermond; Wa = Wageningen). Rarity indicates the rarity of the species according to waarneming.nl (1 = common, 2 = relatively common, 3 = rare, 4 = very rare). Affinity indicates the habitat affinity group as by table A.1 of Turin et al. (2022), a * here indicates that species are more eurytopic. [file elife-104762-supp2.docx]

**Supplementary file 2.** Total number of ground beetles caught per species (or genus), per location. The number of yearseries is given per location in brackets. For some locations, ground beetles were identified up to genus level, these are underlined. “N/A” indicates that this taxa was identified to a different taxonomic level for the specific location. Locations are indicated with abbreviations (Al=Almere; Le = Lelystad; Va = Valthermond; Wa = Wageningen). Rarity indicates the rarity of the species according to waarneming.nl (1 = common, 2 = relatively common, 3 = rare, 4 = very rare). Affinity indicates the habitat affinity group as by table A.1 of Turin et al. (2022), a * here indicates that species are more eurytopic.

|  | **Species** | **Rarity** | **Affinity** | **Al**  **(183)** | **Le**  **(27)** | **Va**  **(72)** | **Wa**  **(179)** | **Total** |
| --- | --- | --- | --- | --- | --- | --- | --- | --- |
|  | **All Carabidae** |  |  | **40153** | **3777** | **1126** | **3052** | **48108** |
|  | **Total Amara** |  |  | **171** | **0** | **27** | **392** | **590** |
| 1 | *Amara aenea* | 1 | Grassland* | 15 | 0 | 8 | 163 | 186 |
| 2 | *Amara anthobia* | 2 | Forest* | 0 | 0 | 3 | 0 | 3 |
| 3 | *Amara apricaria* | 1 | Ruderal* | 0 | 0 | 0 | 19 | 19 |
| 4 | *Amara aulica* | 2 | Grassland | 1 | 0 | 0 | 1 | 2 |
| 5 | *Amara bifrons* | 2 | Ruderal* | 1 | 0 | 1 | 36 | 38 |
| 6 | *Amara communis* | 1 | Grassland* | 0 | 0 | 0 | 1 | 1 |
| 7 | *Amara consularis* | 2 | Ruderal | 0 | 0 | 0 | 54 | 54 |
| 8 | *Amara familiaris* | 1 | Grassland* | 124 | 0 | 0 | 5 | 129 |
| 9 | *Amara fulva* | 2 | Ruderal | 0 | 0 | 6 | 97 | 103 |
| 10 | *Amara ovata* | 2 | Grassland | 0 | 0 | 9 | 0 | 9 |
| 11 | *Amara plebeja* | 1 | Heathland* | 1 | 0 | 0 | 0 | 1 |
| 12 | *Amara similata* | 1 | Ruderal* | 24 | 0 | 0 | 4 | 28 |
| 13 | *Amara spreta* | 1 | Dunes* | 0 | 0 | 0 | 11 | 11 |
| 14 | *Amara tibialis* | 2 | Grassland* | 0 | 0 | 0 | 1 | 1 |
|  | *Amara sp.* |  |  | 5 | N/A | N/A | N/A | 5 |
|  | **Total Anchomenus** |  |  | **151** | **1** | **161** | **8** | **321** |
| 15 | *Anchomenus dorsalis* | 1 | Ruderal | 151 | 1 | 161 | 8 | 321 |
|  | **Total Bembidion** |  |  | **753** | **407** | **32** | **150** | **1342** |
| 16 | *Bembidion aeneum* | 2 | Wetland* | 0 | 1 | N/A | 0 | 1 |
| 17 | *Bembidion biguttatum* | 1 | Ruderal | 7 | 0 | N/A | 0 | 7 |
| 18 | *Bembidion femoratum* | 1 | Wetland | 0 | 0 | N/A | 30 | 30 |
| 19 | *Bembidion lampros* | 1 | Heathland* | 3 | 1 | N/A | 21 | 25 |
| 20 | *Bembidion lunulatum* | 1 | Ruderal* | 3 | 0 | N/A | 0 | 3 |
| 21 | *Bembidion obtusum* | 2 | Ruderal* | 4 | 0 | N/A | 0 | 4 |
| 22 | *Bembidion proprans* | 1 | Ruderal | 4 | 0 | N/A | 23 | 27 |
| 23 | *Bembidion quadrim.* | 1 | Ruderal | 87 | 8 | N/A | 33 | 128 |
| 24 | *Bembidion tetracolum* | 1 | Ruderal* | 345 | 397 | N/A | 43 | 785 |
|  | *Bembidion sp.* |  |  | 300 | N/A | 32 | N/A | 332 |
|  | **Total Blemus** |  |  | **28** | **45** | **0** | **0** | **79** |
| 25 | *Blemus discus* | 2 | Ruderal | 28 | 45 | 0 | 0 | 79 |
|  | **Total Calathus** |  |  | **0** | **0** | **95** | **203** | **298** |
| 26 | *Calathus cinctus* | 2 | Ruderal* | 0 | 0 | 20 | 73 | 93 |
| 27 | *Calathus erratus* | 2 | Heathland* | 0 | 0 | 6 | 87 | 93 |
| 28 | *Calathus fuscipes* | 1 | Grassland* | 0 | 0 | 2 | 0 | 2 |
| 29 | *Calathus melanocephalus* | 1 | Heathland* | 0 | 0 | 67 | 41 | 108 |
| 30 | *Calathus rotundicollis* | 2 | Forest | 0 | 0 | 0 | 2 | 2 |
|  | **Total Clivina** |  |  | **54** | **11** | **4** | **62** | **131** |
| 31 | *Clivina collaris* | 1 | Ruderal* | 0 | 2 | 2 | 28 | 32 |
| 32 | *Clivina fossor* | 1 | Ruderal* | 49 | 9 | 2 | 34 | 94 |
|  | *Clivina sp.* |  |  | 5 | N/A | N/A | N/A | 5 |
|  | **Total Harpalus** |  |  | **2496** | **19** | **481** | **1161** | **4157** |
| 33 | *Harpalus affinis* | 1 | Ruderal* | 131 | 3 | 1 | 30 | 165 |
| 34 | *Harpalus distinguendus* | 2 | Ruderal | 0 | 0 | N/A | 2 | 2 |
| 35 | *Harpalus griseus* | 2 | Forest | 9 | 0 | 2 | 4 | 15 |
| 36 | *Harpalus rubripes* | 2 | Grassland | 0 | 1 | N/A | 1 | 2 |
| 37 | *Harpalus rufipes* | 1 | Ruderal* | 1409 | 15 | 168 | 1104 | 2696 |
| 38 | *Harpalus signaticornis* | 4 | Grassland | 0 | 0 | N/A | 5 | 5 |
| 39 | *Harpalus tardus* | 1 | Grassland* | 0 | 0 | 2 | 15 | 17 |
|  | *Harpalus sp.* |  |  | 947 | N/A | 308 | N/A | 1255 |
|  | **Total Loricera** |  |  | **22** | **6** | **20** | **21** | **69** |
| 40 | *Loricera pilicornis* | 1 | Ruderal* | 22 | 6 | 20 | 21 | 69 |
|  | **Total Nebria** |  |  | **362** | **2** | **0** | **33** | **397** |
| 41 | *Nebria brevicollis* | 1 | Forest* | 320 | 2 | 0 | 16 | 338 |
| 42 | *Nebria salina* | 2 | Heathland | 0 | 0 | 0 | 17 | 17 |
|  | *Nebria sp.* |  |  | 42 | N/A | N/A | N/A | 42 |
|  | **Total Poecilus** |  |  | **4649** | **235** | **82** | **49** | **5015** |
| 43 | *Poecilus cupreus* | 1 | Ruderal | 2876 | 233 | N/A | 24 | 3133 |
| 44 | *Poecilus versicolor* | 1 | Heathland | 4 | 2 | N/A | 25 | 32 |
|  | *Poecilus sp.* |  |  | 1769 | N/A | 82 | N/A | 1851 |
|  | **Total Pterostichus** |  |  | **31041** | **2968** | **217** | **769** | **34995** |
| 45 | *Pterostichus anthracinus* | 2 | Forest | 1 | 0 | N/A | 0 | 1 |
| 46 | *Pterostichus melanarius* | 1 | Ruderal | 5556 | 2731 | 144 | 763 | 9193 |
| 47 | *Pterostichus niger* | 1 | Heathland* | 167 | 228 | 5 | 1 | 401 |
| 48 | *Pterostichus strenuus* | 1 | Forest* | 2 | 0 | N/A | 0 | 2 |
| 49 | *Pterostichus vernalis* | 1 | Ruderal* | 57 | 9 | N/A | 5 | 73 |
|  | *Pterostichus sp.* |  |  | 25258 | N/A | 68 | N/A | 25326 |
|  | **Total Trechus** |  |  | **374** | **76** | **0** | **143** | **593** |
| 50 | *Trechus obtusus* | 1 | Grassland* | 2 | 0 | 0 | 18 | 20 |
| 51 | *Trechus quadristriatus* | 1 | Ruderal* | 69 | 76 | 0 | 125 | 270 |
|  | *Trechus sp.* |  |  | 303 | N/A | N/A | N/A | 303 |
|  | **Total other Carabids** |  |  | **52** | **7** | **7** | **61** | **127** |
| 52 | *Acupalpus meridianus* | 2 | Ruderal | 8 | 3 | 0 | 1 | 12 |
|  | *Acupalpus sp.* |  |  | 2 | N/A | N/A | N/A | 2 |
| 53 | *Agonum muelleri* | 1 | Ruderal | 5 | 3 | 7 | 5 | 20 |
| 54 | *Anisodactylus binotatus* | 1 | Ruderal* | 6 | 0 | 0 | 1 | 7 |
| 55 | *Badister bullatus* | 1 | Dunes | 2 | 0 | 0 | 1 | 3 |
| 56 | *Badister sodalis* | 2 | Heathland | 3 | 0 | 0 | 0 | 3 |
| 57 | *Bradycellus harpalinus* | 1 | Heathland | 1 | 0 | 0 | 0 | 1 |
| 58 | *Broscus cephalotes* | 1 | Dunes | 0 | 0 | 0 | 35 | 35 |
| 59 | *Carabus granulatus* | 1 | Heathland | 2 | 0 | 0 | 0 | 2 |
|  | *Carabus sp.* |  |  | 1 | N/A | N/A | N/A | 1 |
| 60 | *Dyschirius globosus* | 1 | Heathland | 0 | 0 | 0 | 10 | 10 |
| 61 | *Microlestes minutulus* | 3 | Dunes* | 0 | 0 | 0 | 1 | 1 |
| 62 | *Notiophilus aquaticus* | 1 | Heathland | 0 | 0 | 0 | 1 | 1 |
| 63 | *Notiophilus palustris* | 1 | Grassland* | 2 | 0 | 0 | 0 | 2 |
| 64 | *Oxypselaphus obscurus* | 1 | Heathland | 3 | 0 | 0 | 0 | 3 |
| 65 | *Stenolophus teutonus* | 1 | Ruderal* | 0 | 0 | 0 | 2 | 2 |
| 66 | *Stomis pumicatus* | 1 | Forest | 3 | 0 | 0 | 0 | 3 |
| 67 | *Syntomus foveatus* | 1 | Dunes* | 0 | 0 | 0 | 1 | 1 |
| 68 | *Trechoblemus micros* | 2 | Ruderal | 4 | 0 | 0 | 0 | 4 |
|  | Unknown carabidae |  |  | 10 | 1 | 0 | 3 | 14 |
|  |  |  |  |  |  |  |  |  |
