# Supplementary material for: Strip cropping shows promising increases in ground beetle community diversity compared to monocultures
Source: eLife. 2025 Sep 23;14:RP104762. doi: 10.7554/eLife.104762 (PMC12456951; doi:10.7554/eLife.104762)
Supplement: Supplementary file 3. — Results from permanova analyses using Hellinger’s transformation for data from the three locations with species-level data. ‘Crop species’ is a nested variable within years, as these differed among years. Years were nested in locations, as the years that were studied differed among locations. p-values in bold indicate significant effects (α = 0.05). [file elife-104762-supp3.docx]

**Supplementary file 3. Effect of crop configuration on ground beetle community composition.** Results from permanova analyses using Hellinger’s transformation for data from the three locations with species level data. “Crop species” is a nested variable within years, as these differed among years. Years were nested in locations, as the years that were studied differed among locations. P-values in bold typeset indicate significant effects (α = 0.05).

| Location | Predictor | Df | Sum  Sq | R2 | F | P |
| --- | --- | --- | --- | --- | --- | --- |
| Almere | Crop configuration | 1 | 0.20 | 0.01 | 2.34 | **0.025** |
|  | Year | 1 | 2.48 | 0.13 | 28.6 | **0.001** |
|  | Year : Crop species | 14 | 5.66 | 0.30 | 4.68 | **0.001** |
|  | Crop configuration : Year | 1 | 0.13 | 0.01 | 1.50 | 0.138 |
|  | Crop configuration : Year : Crop species | 14 | 2.88 | 0.15 | 2.38 | **0.001** |
|  | *Residual* | 88 | *7.61* | *0.40* |  |  |
|  | *Total* | 119 | *19.0* | *1.00* |  |  |
| Lelystad | Crop configuration | 1 | 0.03 | 0.01 | 0.68 | 0.534 |
|  | Year | 2 | 1.78 | 0.55 | 18.9 | **0.001** |
|  | Year : Crop species | 1 | 0.12 | 0.04 | 2.45 | 0.082 |
|  | Crop configuration : Year | 2 | 0.26 | 0.08 | 2.80 | **0.026** |
|  | Crop configuration : Year : Crop species | 1 | 0.14 | 0.04 | 2.91 | **0.039** |
|  | *Residual* | *19* | *0.90* | *0.28* |  |  |
|  | *Total* | *26* | *3.22* | *1.00* |  |  |
| Wageningen | Crop configuration | 1 | 1.48 | 0.02 | 4.30 | **0.001** |
|  | Year | 3 | 6.40 | 0.07 | 6.22 | **0.001** |
|  | Year : Crop species | 16 | 27.7 | 0.29 | 5.05 | **0.001** |
|  | Crop configuration : Year | 3 | 1.90 | 0.02 | 1.85 | **0.006** |
|  | Crop configuration : Year : Crop species | 16 | 11.8 | 0.12 | 2.15 | **0.001** |
|  | *Residual* | *138* | *47.3* | *0.49* |  |  |
|  | *Total* | *177* | *96.6* | *1.00* |  |  |
