# Supplementary material for: Strip cropping shows promising increases in ground beetle community diversity compared to monocultures
Source: eLife. 2025 Sep 23;14:RP104762. doi: 10.7554/eLife.104762 (PMC12456951; doi:10.7554/eLife.104762)
Supplement: Supplementary file 4. — Results from pairwise permanova analyses for crop pairs pumpkin-barley, cabbage-oat, and potato-grass in 2021 (a) and 2022 (b) in Wageningen. Values show F-values for the comparison between the crop configurations and crops in crossing rows and columns. Bold numbers indicate significant differences between combinations of crop configurations and crops (α = 0.05). [file elife-104762-supp4.docx]

**Supplementary file 4. Effect of crop configuration and crop species on ground beetle community composition.** Results from pairwise permanova analyses for crop pairs pumkin-barley, cabbage-oat, and potato-grass in 2021 (a) and 2022 (b) in Wageningen. Values show F-values for the comparison between the crop configurations and crops in crossing rows and columns. Bold numbers indicate significant differences between combinations of crop configurations and crops (α = 0.05).

**Supplementary file 4a.** 2021

| F-value | | Pumpkin | | Barley | |
| --- | --- | --- | --- | --- | --- |
|  |  | Mono | Strip | Mono | Strip |
| Pumpkin | Mono |  |  |  |  |
|  | Strip | **3.64** |  |  |  |
| Barley | Mono | **10.39** | 1.40 |  |  |
|  | Strip | **5.82** | 1.21 | **4.23** |  |

| F-value | | Potato | | Grass | |
| --- | --- | --- | --- | --- | --- |
|  |  | Mono | Strip | Mono | Strip |
| Potato | Mono |  |  |  |  |
|  | Strip | **5.00** |  |  |  |
| Grass | Mono | 1.70 | 0.57 |  |  |
|  | Strip | **8.38** | 5.06 | 1.94 |  |

| F-value | | Cabbage | | Oat | |
| --- | --- | --- | --- | --- | --- |
|  |  | Mono | Strip | Mono | Strip |
| Cabbage | Mono |  |  |  |  |
|  | Strip | 1.47 |  |  |  |
| Oat | Mono | **2.08** | **3.52** |  |  |
|  | Strip | 2.06 | 2.07 | 2.92 |  |

**Supplementary file 4b.** 2022

| F-value | | Pumpkin | | Barley | |
| --- | --- | --- | --- | --- | --- |
|  |  | Mono | Strip | Mono | Strip |
| Pumpkin | Mono |  |  |  |  |
|  | Strip | 1.42 |  |  |  |
| Barley | Mono | **3.84** | **2.90** |  |  |
|  | Strip | 1.82 | 2.18 | 0.76 |  |

| F-value | | Potato | | Grass | |
| --- | --- | --- | --- | --- | --- |
|  |  | Mono | Strip | Mono | Strip |
| Potato | Mono |  |  |  |  |
|  | Strip | 1.42 |  |  |  |
| Grass | Mono | **7.14** | 3.26 |  |  |
|  | Strip | **4.01** | 3.24 | 3.58 |  |

| F-value | | Cabbage | | Oat | |
| --- | --- | --- | --- | --- | --- |
|  |  | Mono | Strip | Mono | Strip |
| Cabbage | Mono |  |  |  |  |
|  | Strip | **5.35** |  |  |  |
| Oat | Mono | **8.72** | **1.82** |  |  |
|  | Strip | 1.59 | 3.56 | **5.05** |  |
