# Supplementary material for: Strip cropping shows promising increases in ground beetle community diversity compared to monocultures
Source: eLife. 2025 Sep 23;14:RP104762. doi: 10.7554/eLife.104762 (PMC12456951; doi:10.7554/eLife.104762)
Supplement: Supplementary file 5. — Yield results were retrieved from published and unpublished studies on effects of strip cropping on crop yield in similar locations, years, and crops as this study. Mean crop yield is presented in ton per hectare (t/ha). When known, standard deviations of mean crop yield are given (± SD). When a crop is indicated with NC (not collected), the crop yield was not collected due to an inconsistent sampling method (potato, 2020, Almere), crop failure (broccoli, 2020, Almere; celeriac, 2021, Almere), unavailable machine-harvest data (grass, Almere, 2020, 2021, 2022), and undocumented reasons (barley/beans, 2020, Valthermond). Unavailable data include cabbage (2019) and potato (2020) in Lelystad; and pumpkin (2020, 2021, 2022), barley (2020, 2021, 2022), oat (2021, 2022), potato (2021, 2022), grass (2021, 2022), and cabbage (2022) in Wageningen. [file elife-104762-supp5.docx]

**Supplementary file 5. Effect of crop configuration on crop yield.** Yield results were retrieved from published and unpublished studies on effects of strip cropping on crop yield in similar locations, years, and crops as this study. Mean crop yield is presented in ton per hectare (t/ha). When known, standard deviations of mean crop yield are given (± SD). When a crop is indicated with NC (not collected) the crop yield was not collected due to an inconsistent sampling method (potato, 2020, Almere), crop failure (broccoli, 2020, Almere; celeriac, 2021, Almere), unavailable machine-harvest data (grass, Almere, 2020, 2021, 2022) and undocumented reasons (barley/beans, 2020, Valthermond). Unavailable data include cabbage (2019) and potato (2020) in Lelystad; and pumpkin (2020, 2021, 2022), barley (2020, 2021, 2022), oat (2021, 2022), potato (2021, 2022), grass (2021, 2022), and cabbage (2022) in Wageningen.

| Location | Year | Crops | Reference | Yield (t/ha) |  |
| --- | --- | --- | --- | --- | --- |
|  |  |  |  | Mono | Strip |
| Almere | 2020 | Beans | Juventia et al., 2024 | 2.80 | 3.45 |
|  |  | Broccoli |  | NC | NC |
|  |  | Celeriac |  | 54.44 | 69.57 |
|  |  | Grass |  | NC | NC |
|  |  | Oat |  | 6.05 | 5.08 |
|  |  | Onion |  | 41.06 | 42.14 |
|  |  | Parsnip |  | 27.50 | 35.36 |
|  |  | Potato |  | NC | NC |
|  | 2021 | Beans | Juventia et al., 2024 | 4.72 | 6.50 |
|  |  | Broccoli |  | 9.30 | 4.90 |
|  |  | Celeriac |  | NC | NC |
|  |  | Grass |  | NC | NC |
|  |  | Oat |  | 8.76 | 8.25 |
|  |  | Onion |  | 39.03 | 37.39 |
|  |  | Parsnip |  | 36.88 | 65.01 |
|  |  | Potato |  | 24.68 | 27.88 |
|  | 2022 | Beans | Juventia et al., 2024 | 4.12 | 4.36 |
|  |  | Broccoli |  | 1.12 | 1.59 |
|  |  | Celeriac |  | 45.38 | 51.45 |
|  |  | Grass |  | NC | NC |
|  |  | Oat |  | 6.27 | 6.70 |
|  |  | Onion |  | 57.89 | 48.62 |
|  |  | Parsnip |  | 20.6 | 21.09 |
|  |  | Potato |  | 31.08 | 31.60 |
| Lelystad | 2020 | Cabbage | Carillo-Reche et al., 2023 | 44.0 ± 12.8 | 46.3 ± 13.0 |
|  | 2021 | Cabbage | Carillo-Reche et al., 2023 | 85.6 ±18.2 | 75.9 ± 23.4 |
| Valthermond | 2020 | Barley | Unpublished data | 5.3 | 5.1 |
|  |  | Barley / Beans |  | NC | 5.1 |
|  |  | Potato |  | 20.9 | 20.5 |
|  | 2021 | Barley / Beans | Unpublished data | 3.1 | 3.4 |
|  |  | Grass / Clover |  | 8.3 | 7.9 |
|  |  | Potato |  | 23.7 | 20.7 |
| Wageningen | 2019 | Cabbage | Carillo-Reche et al., 2023 | 36.8 ± 15.8 | 34.6 ± 6.5 |
|  |  | Wheat | Ditzler et al., 2023 | 2.8 ± 0.2 | 2.1 ± 0.3 |
|  |  | Potato | Ditzler et al., 2023 | 29.3 ± 5.7 | 30.6 ± 8.8 |
|  | 2020 | Cabbage | Carillo-Reche et al., 2023 | 25.7 ± 9.3 | 19.3 ± 6.6 |
|  |  | Wheat | Ditzler et al., 2023 | 0.9 ± 0.2 | 1.0 ± 0.4 |
|  |  | Potato | Ditzler et al., 2023 | 23.4 ± 6.1 | 26.1 ± 8.9 |
|  | 2021 | Cabbage | Carillo-Reche et al., 2023 | 32.1 ± 7.1 | 27.9 ± 13.1 |
