# Supplementary material for: Strip cropping shows promising increases in ground beetle community diversity compared to monocultures
Source: eLife. 2025 Sep 23;14:RP104762. doi: 10.7554/eLife.104762 (PMC12456951; doi:10.7554/eLife.104762)
Supplement: Supplementary file 6. — Rounds indicate the number of times pitfall traps were placed and were pooled within year series. [file elife-104762-supp6.docx]

**Supplementary file 6. Sampling effort.** The total numbers of pitfall traps placed per location, year and crop. Rounds indicate the number of times pitfall traps were placed and were pooled within year series.

| Location | Year | Crops | Rounds | Pitfall traps per round | |
| --- | --- | --- | --- | --- | --- |
|  |  |  |  | Mono | Strip |
| Almere | 2020 | Beans | 3 | 4 | 4 |
|  |  | Broccoli |  | 4 | 4 |
|  |  | Celeriac |  | 4 | 4 |
|  |  | Grass |  | 4 | 3 |
|  |  | Oat |  | 4 | 4 |
|  |  | Onion |  | 4 | 4 |
|  |  | Parsnip |  | 4 | 4 |
|  |  | Potato |  | 4 | 4 |
|  | 2021 | Beans | 4 | 4 | 4 |
|  |  | Broccoli |  | 4 | 4 |
|  |  | Celeriac |  | 4 | 4 |
|  |  | Grass |  | 3 | 4 |
|  |  | Oat |  | 4 | 4 |
|  |  | Onion |  | 4 | 4 |
|  |  | Parsnip |  | 4 | 4 |
|  |  | Potato |  | 4 | 4 |
|  | 2022 | Beans | 5 | 4 | 4 |
|  |  | Broccoli |  | 4 | 3 |
|  |  | Celeriac |  | 3 | 2 |
|  |  | Grass |  | 4 | 4 |
|  |  | Oat |  | 4 | 2 |
|  |  | Onion |  | 3 | 4 |
|  |  | Parsnip |  | 4 | 4 |
|  |  | Potato | 4 | 4 | 4 |
| Lelystad | 2019 | Cabbage | 5 | 4 | 3 |
|  | 2020 | Cabbage | 7 | 4 | 3 |
|  |  | Potato | 2 | 4 | 2 |
|  | 2021 | Cabbage | 6 | 4 | 3 |
| Valthermond | 2020 | Barley | 7 | 6 | 6 |
|  |  | Barley / Beans |  | 6 | 6 |
|  |  | Potato |  | 6 | 6 |
|  | 2021 | Barley / Beans | 8 | 6 | 6 |
|  |  | Grass / Clover |  | 6 | 6 |
|  |  | Potato |  | 6 | 6 |
| Wageningen | 2019 | Cabbage | 4 | 6 | 2 |
|  |  | Wheat | 2 | 6 | 2 |
|  |  | Potato | 3 | 6 | 6 |
|  | 2020 | Cabbage | 8 | 6 | 2 |
|  |  | Wheat | 3 | 5 | 2 |
|  |  | Potato | 1 | 6 | 5 |
|  |  | Pumpkin | 2 | 6 | 4 |
|  |  | Barley |  | 6 | 2 |
|  | 2021 | Cabbage | 7 | 6 | 3 |
|  |  | Oat |  | 6 | 3 |
|  |  | Potato | 2 | 6 | 3 |
|  |  | Grass |  | 6 | 3 |
|  |  | Pumpkin |  | 6 | 3 |
|  |  | Barley |  | 6 | 3 |
|  | 2022 | Cabbage | 2 | 6 | 3 |
|  |  | Oat |  | 5 | 3 |
|  |  | Potato |  | 6 | 3 |
|  |  | Grass |  | 6 | 3 |
|  |  | Pumpkin |  | 6 | 3 |
|  |  | Barley |  | 6 | 3 |
