# Supplementary material for: Strip cropping shows promising increases in ground beetle community diversity compared to monocultures
Source: eLife. 2025 Sep 23;14:RP104762. doi: 10.7554/eLife.104762 (PMC12456951; doi:10.7554/eLife.104762)
Supplement: Supplementary file 7. [file elife-104762-supp7.docx]

**Supplementary file 7.** **Plant species composition of flower strips** adjacent to the strip cropping fields in Lelystad and Valthermond.

|  | **Species** |
| --- | --- |
| 1 | *Achillea millefolium* (common yarrow) |
| 2 | *Anethum graveolens* (dill) |
| 3 | *Cichorium intybus* (common chichory) |
| 4 | *Foeniculum vulgare* (fennel) |
| 5 | *Pastinaca sativa* (parsnip) |
| 6 | *Angelica sylvestris* (wild angelica) |
| 7 | *Anthriscus sylvestris* (cow parsley) |
| 8 | *Fagopyrum esculentum* (buckwheat) |
| 9 | *Papaver Rhoeas* (common poppy red wild form) |
| 10 | *Chrysanthemum segetum* (corn marigold) |
| 11 | *Ammi majus* (greater ammi) |
| 12 | *Centaurea cyanus* (cornflower, single flowered wild form) |
